# Effect of *In Vitro* Maturation Technique and Alpha Lipoic Acid Supplementation on Oocyte Maturation Rate: Focus on Oxidative Status of Oocytes

**DOI:** 10.22074/ijfs.2015.4601

**Published:** 2015-12-23

**Authors:** Saeed Zavareh, Isaac Karimi, Mojdeh Salehnia, Ali Rahnama

**Affiliations:** 1 School of Biology, Damghan University, Damghan, Iran; 2 Institute of Biological Sciences, Damghan University, Damghan, Iran; 3 Laboratory of Molecular and Cellular Biology, Department of Basic Veterinary Sciences, School of Veterinary Medicine, Razi University, Kermanshah, Iran; 4 Department of Anatomy, Faculty of Medical Sciences, Tarbiat Modares University, Tehran, Iran

**Keywords:** Oocytes Maturation, Oxidative Statuse, Alpha Lipoic Acid

## Abstract

**Background:**

Therapeutic potential of *in vitro* maturation (IVM) in infertility is growing with great promise. Although significant progress is obtained in recent years, existing IVM protocols are far from favorable results. The first aim of this study was to investigate whether two step IVM manner change reactive oxygen species (ROS) and total anti- oxidant capacity (TAC) levels. The second aim was to find the effect of alpha lipoic acid (ALA) supplementation on oocyte maturation rate and on ROS/TAC levels during IVM.

**Materials and Methods:**

In this experimental study, mouse germinal vesicle (GV) oocytes divided into cumulus denuded oocytes (DOs) and cumulus oocyte complexes (COCs) groups. GVs were matured *in vitro* in the presence or absence of ALA only for 18 hours (control) or with pre-culture of forskolin plus cilostamide for an additional 18 hours. Matured oocytes obtained following 18 and 36 hours based on experimental design. In parallel, the ROS and TAC levels were measured at different time (0, 18 and 36 hours) by 2',7'-dichlorodihydrofluorescein (DCFH) probe and ferric reducing/antioxidant power (FRAP) assay, respectively.

**Results:**

Maturation rate of COCs was significantly higher than DOs in control group (P<0.05), while there was no significant difference between COCs and DOs when were pre-cultured with forskolin plus cilostamide. ROS and TAC levels was increased and decreased respectively in DOs after 18 hours while in COCs did not change at 18 hours and showed a significant increase and decrease respectively at 36 hours (P<0.05). ROS and TAC levels in the presence of ALA were significantly decreased and increased respectively after 36 hours (P<0.05) whereas, maturation rates of COCs and DOs were similar to their corresponding control groups.

**Conclusion:**

ALA decreased ROS and increased TAC but could not affect maturation rate of both COCs and DOs in one or two step IVM manner.

## Introduction

*In vivo* maturation (IVM) of oocytes is an important issue of assisted reproduction techniques (ART) which is used as a method in treatment of infertility. The success of IVM requires a fine synchrony between nuclear maturation and cytoplasmic maturation (1). In the *in vitro* condition, cytoplasmic maturation of oocyte lags behind since cytoplasmic maturation completes gradually during folliculogenesis therefore an asynchrony is occurred between nuclear maturaion and cytoplasmic maturation (1,2). 

It has been revealed that higher cellular cyclic adenosine mono-phosphate (cAMP) level could arrest the oocyte nuclear maturation and improve oocyte development competence (3,4) by providing an opportunity for essential modification of cellular organelles and biochemical state to sustain normal fertilization and further embryonic development (5). 

Adenylyl cyclase (AC) and phosphodiesterases (PDEs) are two enzymes that control intra-oocyte level of cAMP via its synthesis and degradation, respectively (6). cAMP-dependent protein kinases through inhibiting of maturation-promoting factor (MPF) and mitogenactivating protein kinase (MAPK) arrest the meiotic division (7). Hence, it has been postulated that adding an AC activator and/or PDEs inhibitors to the oocytes maturation medium via increasing cAMP level prevents nuclear maturation and consequently, the maturation of oocyte cytoplasm and nucleus will be somewhat concurrent (3,8,12). It has been demonstrated that cumulus cells produce cAMP and transmits it via gap junctions to oocyte which in turn supporting nuclear and cytoplacmic maturation of oocyte (13). 

Higher oxygen concentration in the *in vitro* systems than *in vivo* condition lead to build up of reactive oxygen species (ROS) (14). Amongst numerous factors that may be contributed in low outcomes of IVM, production of ROS within the oocytes and culture medium lead to oxidative stress (OS) that affects IVM. However, the role of ROS in IVM of oocyte and its developmental competence remains controversial. Under optimal *in vivo* condition, increased generation of ROS was neutralized by both enzymatic and non-enzymatic antioxidants (15). It has been shown that, total antioxidant capacity (TAC) of ovarian follicular fluid can serve as a predictive marker of *in vitro* fertilization (IVF) success (16). Therefore, there is an urgent need to understand the scope of the OS-relevant factors that may affect oocyte development. In this continuum, supplementing maturation medium with different antioxidants has been reported that overcome OS and improves oocyte developmental competence (15,17,20). In this sense, alpha lipoic acid (ALA) as a component of biological membranes and an imperative cofactor of mitochondrial dehydrogenases is well known for its antioxidative properties (21,22). The ALA and its reduced form dihydrolipoic acid (DHLA), has been shown as potent antioxidant in both *in vivo* (23,25) and *in vitro* conditions (17). In essence, due to the unfavorable outcome of IVM and suspicious effects of OS on the oocyte IVM, the purposes of the present study were i. Assessment of the changes of ROS and TAC levels during two step IVM of oocytes with or without cumulus cells and ii. Determine whether adding ALA to maturation medium considering increasing cultivation period and possibility of excessive production of ROS in two step culture manner could modify ROS and TAC levels and improve the oocyte maturation. 

## Materials and Methods

### Reagents

All chemicals were purchased from SigmaAldrich, UK unless otherwise stated and all media were prepared using Milli-Q water. 

## Animal subjects

In this experimental animal study, adult female (8-10 weeks old, n=25) Naval Medical Research Institute (NMRI) mice supplied from Pasteur Institute, Iran were cared for and used according to the guide for the care and use of laboratory animals of our university that fulfills and follows declaration of Helsinki as revised in Tokyo 2004. They were housed under 12 hours light: 12 hours dark regimen (light on at 7:00 am), at temperature of 23 ± 3˚C and relative humidity of 44 ± 2% for at least 1 week before use with free access to food and water. 

## Experimental design

Germinal vesicle (GV) oocytes (vide infra) were divided into two main groups: cumulus oocyte complexes (COCs) and cumulus denuded (DOs). Each main group was randomly distributed among following subgroups: i. IVM without any intervention (control) and ii. IVM in the presence of 50 µM forskolin, an AC activator, in combination with 10 µM cilostamide, a PDE3 inhibitor. In separate experiments, each group was also cultured in the presence or absence of ALA. In sum, 8 experimental groups were studied. IVM with meiotic inhibitors were performed in two step manner, briefly; step i. GV oocytes were cultured with cilostamide and forskolin for 18 hours and then step ii. Meiotic inhibitors were removed and oocytes were cultured for additional 18 hours. The control group was cultured without any meiotic inhibitors only for 18 hours (one step culture). Based on the experimental group, 18 or 36 hours after onset of cultivation, the maturational status of the oocytes in the each group was examined and classified as GV, GV breakdown (GVBD) or metaphase II (MII). In parallel, biochemical assay were performed for determining of TAC and ROS levels at initial time, 18 and 36 hours based on the experimental groups. 

## Preparation of cilostamide, forskolin and alpha lipoic acid stock solutions

Cilostamide, forskolin and ALA were dissolved in dimethylesulphoxide (DMSO) at 100 mM concentration as stock solution, protected from light and kept at -20˚C. Before using, they were immediately diluted to the appropriate concentration in maturation medium to reach the final concentrations of 10, 50 and 100 µM respectively. Final concentrations of DMSO in maturation medium were 0.001% for cilostamide, 0.005% for forskolin and 0.01% for ALA. It has been shown that, the concentration of DMSO in the medium up to 0.1%, does not have any adverse effect on oocyte (4). 

## Isolation of germinal vesicle oocytes

GV oocytes were obtained from 8-10 weeks old female mice based on described previously methods (26). Briefly, primed mice with an intraperitoneal injection of 7.5 IU pregnant mare’s serum gonadotropin (PMSG, Intervet, Australia) were killed after 48 hours by cervical dislocation and their ovaries were collected in HEPES-buffered TCM199 medium (Gibco, UK) supplemented with 10% (v/v) fetal bovine serum (FBS, Gibco, UK), 0.23 mM sodium pyruvate, 100 IU/ml penicillin and 75 µg/ml streptomycin. The COCs were achieved by puncture of antral follicles with sterile 29 gauge needles. COCs with uniform covering of 3-5 layers of cumulus cells and homogenous cytoplasm were selected for experiments. The DOs were obtained by repeated pipetting and flushing a portion of COCs through a small fine controlled bore pipette. After washing the oocytes in fresh HEPES–buffered TCM199 medium; they were used as described in experimental design. Collections of oocytes were performed at minimum possible time prior transfer to maturation medium. 

Also, in order to prevent spontaneous maturation, oocytes of each experimental group were collected in the presence of meiotic inhibitors which used in the same group. 

## *In vitro* maturation of germinal vesicle oocytes

The basal maturation medium was as described previously (27). In brief, TCM199 supplemented with 0.22 g/L NaHCO_3_ 100 IU/ml penicillin and 75 μg/ml streptomycin, 0.23 mM sodium pyruvate, 10% FBS, 10 ng/ml epidermal growth factor (EGF), 75 mIU/ml recombinant human follicle stimulating hormon (rhFSH) and 10 IU/ml human chorionic gonadotropin (hCG). According to the experimental design, 100 µM of ALA, 10 µM of cilostamide and 50 µM of forskolin were added to the maturation medium. Groups of five oocytes were cultured in a drop of 20 µl of maturation medium under mineral oil at 37˚C, 100% humidity in 5% CO_2_ forfor 18 or 36 hours according to experimental design. At the end of the culture period, the number of degenerated oocytes, oocytes at GV, GVBD and MII stages were counted using an inverted microscope with Hoffman modulation contrast equipment (Nikon, Japan). 

## Biochemical assay

The intracellular ROS production of cultured oocytes was measured as described previously with some modification (17,18). Briefly, 15 oocytes were used at different times of culturing period, (0, 18 and 36 hours) in each study group. The oocytes were washed with phosphate buffer saline solution (PBS, Gibco, UK) and incubated in 40 mM of Tris–HCl buffer (pH=7.0) containing 5 uM of 2´,7´-dichlorodihydrofluorescin diacetate (DCHFDA, Merck, Germany) at 37˚C for 30 minutes. Oocytes were then washed again with PBS and then transferred to 100 µM of Tris-HCl buffer (40 mM, pH=7.0) and sonicated at 50 W for 1 minute. Resulting mixture was centrifuged at 10000×g for 20 minutes at 4˚C and fluorescent intensity of supernatant was monitored by using a spectrofluorometer at 488 nm excitation and 525 nm emission. Corrections for autofluorescence were made by including parallel blank in each experiment. Standards curves were conducted by using known amounts of H_2_O_2_ as described previously (28).All experiments were repeated at least four times. 

The assessment of TAC levels of cultured oocytes was performed according to common ferric reducing/ antioxidant power (FRAP) method (17,18). Briefly, 15 oocytes at different times of culture period (0, 18 and 36 hours) were homogenized in 100 ul Tris–HCl buffer (40 mM, pH=7.0) and sonicated at 50 W for 1 minute then centrifuged at 10000×g for 20 minutes at 4˚C. Cellular supernatant (100 µl) was added to 1 ml of freshly prepared FRAP reagent (Tripyridyltriazine, TPTZ, Merck, Germany) in a cuvette and incubated in 37˚C for 10 minutes. Absorbance of blue-colored reagent was read against a blank at 593 nm. 

## Statistical analysis

All experiments were repeated at least four times. Differences among groups in the proportion of GV, GVBD and MII oocytes and TAC and ROS levels were statistically analyzed by one-way ANOVA using SPSS (version 19, Chicago, IL, USA) software. An independent sample t test was conducted to compare the rates of meiotic stages (GV, GVBD and MII) and TAC and ROS levels of ALA-treated groups and non-ALA treated groups. Percentages of degeneration, GV, GVBD and MII were statistically analyzed after arcsine transformation. Assessment of interaction among ALA, cumulus cells and meiotic inhibitors were statically analyzed by two-way ANOVA. When ANOVA indicated a significant difference (P<0.05), post hoc Tukey’s HSD was used. 

## Results

### Maturational status of oocytes

Rates of GV, GVBD, MII and degenerated oocytes of COCs and DOs following one step (control) and two step IVM manner in the presence or absence of ALA are shown in table 1. 

**Table 1:** Rates of GV, GVBD. MII and degenerated oocytes of COCs and DOs following one and two step *in vitro* maturation in the presence or absence of ALA


Groups		n	GV		GVBD		MII		Degeneration	
			ALA+	ALA-	ALA+	ALA-	ALA+	ALA-	ALA+	ALA-
			n/all (% ± SD)	n/all (% ± SD)	n/all (% ± SD)	n/all (% ± SD)	n/all (% ± SD)	n/all (% ± SD)	n/all (% ± SD)	n/all (% ± SD)

Control	COC	352	27/120 (22.50 ± 2.50)^a^	54/232 (23.26 ± 0.55)^a^	17/120 (14.17 ± 1.44)^a^	31/232 (14.17 ± 1.44)^a^	73/120^*^ (60.83 ± 1.44)	138/232^*^ (59.51 ± 1.59)	3/120 (2.50 ± 2.50)	9/232 (3.88 ± 0.13)
	DO	176	29/98 (29.59 ± 2.14)^b^	22/78 (28.16 ± 2.07)^b^	20/98 (20.40 ± 0.70)^b^	20/98 (20.40 ± 0.70)^b^	45/98 (45.94 ± 0.64)	33/78 (42.43 ± 2.25)	4/98 (5.19 ± 2.05)	7/78 (8.97 ± 1.38)
Cilostamide and forskolin	COC	240	7/120 (5.83 ± 1.44)^c^	8/120 (6.67 ± 1.44)^c^	32/120 (25.00 ± 2.50)^b^	32/120 (25.00 ± 2.50)^b^	82/120^#^ (68.33 ± 1.44)	80/120^#^ (66.67 ± 1.44)	0/120 (0.00 ± 0.00)	1/120 (0.83 ± 1.44)
	DO	240	13/120 (10.83 ± 1.44)^c^	14/120 (11.67 ± 1.44)^c^	27/120 (22.50 ± 0.00)^b^	27/120 (22.50 ± 0.00)^b^	79/120^#^ (65.83 ± 1.44)	77/120^#^ (64.17 ± 1.44)	1/120 (0.83 ± 1.44)	4/120 (3.33 ± 1.44)


Different superscript letters in the same columns indicate significant differences (P<0.05). ^*^; Indicate significant difference with respective DOs groups, ^#^; Indicate significant difference with respective control group, GV; Germinal vesicle, GVBD; Germinal vesicle breackdown, MII; Metaphase II, COC; Cumulus oocytes complexe, DO; Cumulus denuded oocyte and ALA; Alpha lipoic acid. Two step *in vitro* maturation manner: cultured with meiotic inhibitors from 0 to 18 hours, no meiotic inhibitors from 18 to 36 hours, One step *in vitro* maturation manner (control): cultured without meiotic inhibitors only for 18 hours.

After 18 hours culture in one step IVM manner, the rates of MII oocytes (59.5%), in COCs control group were significantly higher than DOs control group (42.4%). The rates of GV (23.26%), GVBD (13.34%) and degeneration (3.88%) in the COCs control groups were also statistically lower than those of DOs control group (28.16, 20.42 and 8.97% respectively). At end of two step IVM manner, MII rate (66.7%) of COCs was significantly higher than COCs control group. In addition, 6.67% of COCs was arrested at GV stage which was significantly lower than those of COCs control group. On the contrary, the rate of GVBD (25.83%) for two step *in vitro* matured COCs groups was significantly higher than those of COCs control group. The percentages of degenerated oocytes were not statistically different among groups (P<0.05). The rate of MII oocytes in cilostamide and forskolin treated DOs groups was 64.2% which was significantly higher than that of respective control group. The rates of oocytes that arrested at the GV stage after 36 hours culture in cilostamide and forskolin treated DOs group (11.67%) was significantly lower than that of control DOs group. There was no significant difference between GVBD rate of pre-matured DOs group with combination of cilostamide and forskolin (20.63%) and that of control DOs group (P<0.05, Table 1). There was no significant difference between degeneration rates of DOs control group and that of cilostamide and forskolin treated DOs group (P<0.05). A similar rate of MII, GV, GVBD and degeneration were observed in the presence or absence of ALA in all experiment groups (P<.05). 

There were no significant differences in the rates of MII oocytes, of COCs groups after prematuration with forskolin and cilostamide with those of DOs groups (P<0.05) while, the rates of MII oocytes of COCs control groups were statistically higher than those of DOs control groups. There was a significant interaction between the effect of pre-maturation with meiotic inhibitors and the presence and absence of cumulus cells on the rates of MII oocytes. They were more effective on the DOs than COCs groups. Also, there was no interaction between ALA and meiotic inhibitors and presence or absence of cumulus cells (P<0.05). 

## Oxidative status

**Fig.1:**
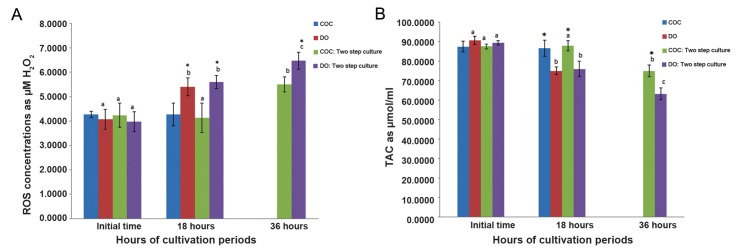
A. ROS and B. TAC concentrations of one and two step *in vitro* cultured oocytes without pre-treatment of ALA. In all cases 4 experimental replicates were performed. Different superscripts (a, b, c) reflect different levels of significant differences at different times of cultivation period within the same group (P<0.05). *; Indicate significant differences with COC groups at same times of cultivation period (P<0.05), ROS; Reactive oxygen species, TAC; Total antioxidant capacity, DO; Denuded oocytes and COC; Cumulus oocyte complexe. Two step *in vitro* maturation manner: cultured with meiotic inhibitors from 0 to 18 hours, no meiotic inhibitors from 18 to 36 hours. One step *in vitro* maturation manner (control): cultured without meiotic inhibitors only for 18 hours. Data were presented as mean ± SE.

ROS concentration and TAC of COCs (4.27 ± 0.13 µM and 87.5 ± 2.78 µmol/ul respectively) and DOs (4.07 ± 0.4 µM and 90.77 ± 2.11 µmol/µl respectively) at initial time of culture period were not significantly different among groups (P<0.05, Fig .1). The ROS concentrations of COCs did not increase significantly at 18 hours (4.27 ± 0.47 µM, Table 2), while ROS production in DOs (5.4 ± 0.36 µM) significantly increased (Fig .1A,Table 2). In two step culture condition a significant increase of ROS level were observed in both COCs and DOs from18 hours (4.13 ± 0.6 µM and 5.6 ± 0.26 µM respectively, Table 3) to 36 hours (5.5 ± 0.3 µM and 6.47 ± 0.35 µM repectively, Fig .1). Also, the ROS levels of DOs at 18 and 36 hours of culture (5.6 ± 0.26 µM and 6.47 ± 0.35 µM respectively, Table 3) were significantly higher than those of COCs (4.13 ± 0.6 µM and 5.5 ± 0.3 µM respectively, Fig .1A). 

**Fig.2:**
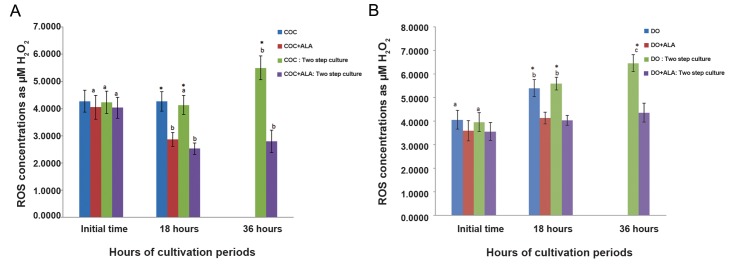
A. ROS concentrations of one and tw o step *in vitro* cultured COCs and B. DOs with or without pr e-treatment of ALA. In all cases 4 experimental replicates were performed. Different superscripts (a, b, c) reflect different levels of significant differences at different times of cultivation period within the same gr oup (P<0.05). ^*^; Indicate significant differences with respective ALA treated groups at same times of cultivation period (P<0.05), ROS; Reactive oxygen species, DO; Denuded oocytes, COC; Cumulus oocyte complexe and ALA; Alpha lipoic acid. Two step *in vitro* maturation manner: cultured with meiotic inhibitors from 0 to 18 hours, no meiotic inhibitors from 18 to 36 hours. One step *in vitro* maturation manner (control): cultured without meiotic inhibitors only for 18 hours. Data were presented as mean ± SE.

In the presence of ALA, ROS production of COCs was significantly decreased from initial time (4.03 ± 0.42 µM) to 18 hours (2.53 ± 0.42 µM, Table 3) and remained without change, up to 36 hours (2.8 ± 0.26 µM) in two step culture condition (P<0.05, Fig .2A). ROS levels of ALA treated DOs did not significantly change during culture period up to 36 hours (initial time: 3.57 ± 0.38 µM, 18 hours: 4.03 ± 0.21 µM, 36: 4.37 ± 0.4 µM, P<0.05, Fig .2B). 

**Fig.3:**
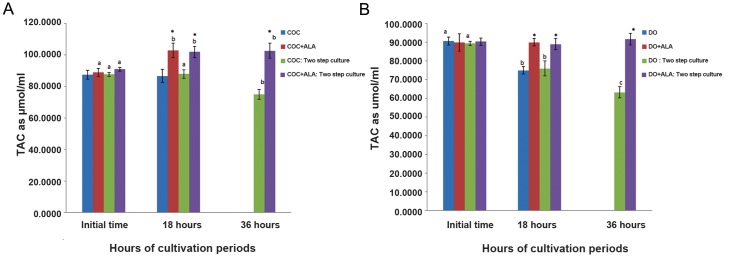
TAC level of one and two step *in vitro* cultured COCs and B. DOs with or without pre-treatment of ALA In all cases 4 experimental replicates were performed. Different superscripts (a, b, c) reflect different levels of significant differences at different times of cultivation period within the same gr oup (P<0.05). *; Indicate significant differences with respective non-ALA treated groups at same times of cultivation period (P<0.05), TAC; Total antioxidant capacity, DO; Denuded oocytes, COC; Cumulus oocyte complexe and ALA; Alpha lipoic acid. Two step *in vitro* maturation manner: cultured with meiotic inhibitors from 0 to 18 hours, no meiotic inhibitors from 18 to 36 hours. One step *in vitro* maturation manner (control): cultured without meiotic inhibitors only for 18 hours. Data were presented as mean ± SE.

As showed in table 3, maximum TAC levels in COCs was observed at the initial time (87.5 ± 2.8 µmol/µl) and 18 hours (86.7 ± 4.2 µmol/ µl), while, significantly decreased up to 36 hours (75.0 ± 3.0 µmol/µl) in two step *in vitro* condition (Fig .3). TAC levels of ALA treated COCs increased significantly after 18 hours (103.0 ± 4.6 µmol/µl, Table 3) of culture and remained constant without significant difference up to 36 hours later (102.7 ± 4.7 µmol/ µl, Fig .3A). In DOs group, TAC levels were significantly decreased at 18 hours (76.0 ± 4.0 µmol/µl) and 36 hours (63.3 ± 3.1 µmol/µl) in comparison with initial time (89.5 ± 1.0 µmol/ µl, Fig .3B). While, there was no significant difference in the TAC levels of ALA treated DOs between the end (91.7 ± 3.1 µmol/µl, Table 3) and beginning (90.3 ± 1.9 µmol/µl) of the culture period (P<0.05, Fig .3B, Table 3). 

**Table 2:** ROS concentrations as µM H_2_O_2_ of one and two step *in vitro* cultured oocytes with or without pre-treatment of ALA


Groups		Initial time		18 hours		36 hours	
		ALA+	ALA-	ALA+	ALA-	ALA+	ALA-

Control	COC	4.05 ± 0.13	4.27 ± 0.13	2.87 ± 0.25^*a^	4.27 ± 0.47	-	-
	DO	3.60 ± 0.44	4.07 ± 0.4	4.13 ± 0.25^#^	5.40 ± 0.36^a^	-	-
Cilostamide and forskolin	COC	4.03 ± 0.42	4.23 ± 0.49	2.53 ± 0.42^*a^	4.13 ± 0.6	2.80 ± 0.26^*a^	5.50 ± 0.30^a^
	DO	3.57 ± 0.38	3.97 ± 0.4	4.03 ± 0.21^*#^	5.60 ± 0.26^#a^	4.37 ± 0.4^*#^	6.47 ± 0.35^a^


In all cases 4 experimental replicates were performed, ^a^; Reflect significant differences with initial time within the same group (P<0.05), ^*^; Indicate significant differences with respective ALA treated groups at same times of cultivation period (P<0.05), ^#^; Indicate significant differences with COC groups at same times of cultivation period (P<0.05), ROS; Reactive oxygen species, DO; Denuded oocytes, COC; Cumulus oocyte complexe and ALA; Alpha lipoic acid. Two step *in vitro* maturation manner: cultured with meiotic inhibitors from 0 to 18 hours, no meiotic inhibitors from 18 to 36 hours, One step *in vitro* maturation manner (control): cultured without meiotic inhibitors only for 18 hours. Data were presented as mean ± SE.

**Table 3:** TAC concentrations as μM/μL of one and two step *in vitro* cultured oocytes with or without pre-treatment of ALA


Groups		Initial time		18 hours		36 hours	
		ALA+	ALA-	ALA+	ALA-	ALA+	ALA-

Control	COC	88.90 ± 2.54	87.50 ± 2.78	103.00 ± 4.58^a^	86.67 ± 4.16^*^	-	-
	DO	90.07 ± 4.61	90.77 ± 2.11	90.00 ± 2.00^#^	75.00 ± 2.0^a#^	-	-
Cilostamide and forskolin	COC	90.93 ± 1.1	87.67 ± 1.26	102.00 ± 3.61^a^	88.00 ± 2.65^*^	102.67 ± 4.73^a^	75.00 ± 3.0^a*^
	DO	90.33 ± 1.9	89.50 ± 1.04	89.00 ± 3.0^#^	76.00 ± 4.0^a*#^	91.67 ± 3.06^#^	63.33 ± 3.06^a*#^


In all cases 4 experimental replicates were performed, a; Reflect significant differences with initial time within the same group (P<0.05), ^*^; Indicate significant differences with respective ALA treated groups at same times of cultivation period (P<0.05), ^#^; Indicate significant differences with COC groups at same times of cultivation period (P<0.05), TAC; Total antioxidant capacity, DO; Denuded oocytes, COC; Cumulus oocyte complexe and ALA; Alpha lipoic acid. Two step *in vitro* maturation manner: cultured with meiotic inhibitors from 0 to 18 hours, no meiotic inhibitors from 18 to 36 hours, One step *in vitro* maturation manner (control): cultured without meiotic inhibitors only for 18 hours. Data were presented as mean ± SE.

## Discussion

In this investigation, cilostamide and forskolin have been used in the oocyte maturation medium to coincide maturation of nuclear and cytoplasm of mouse oocytes. In the recent years, many advantages have been achieved in the oocyte IVM, although oocyte IVM is always associated with many challenges. One of the predicaments is absence of critical cytoplasmic biochemical and molecular events which are essential for acquisition of developmental competence of oocyte (13). Reversible cessation of oocyte meiotic division using cAMP elevating agents in order to improve oocyte developmental competence via synchrony of nuclear and cytoplasmic maturation has been shown previously (3,4,8,9,11,12). In this study, a combination of forskolin and cilostamide was used because it was demonstrated that there is a synergistic effect between those on the oocyte maturation, fertilization and subsequent embryonic development (12). The role of delayed *in vitro* oocyte nuclear maturation in improvement of developmental competence remains controversial. Some studies confirmed that postponement of oocyte nuclear maturation leads to increase developmental failure (29) while others believe that it progressed developmental competence through improvement of oocyte cytoplasmic and nuclear maturation (3,9,11,12). However, our findings indicate that combination of forskolin and cilostamide will improve developmental competence of mouse oocytes. 

This study showed higher maturation rate of COCs than DOs in one-step IVM manner while there were no significant difference between maturation rates of COCs and DOs in biphasic IVM manner. In this line, several studies have showed the decisive role of cumulus cells in the oocyte developmental competence (23,30). These studies indicate that meiosis inhibitors were more effective on the DOs than COCs in two-step IVM manner. It seems that exposure to gonadotrophin in the absence of cumulus cells accelerate meiotic progression as a non-physiological condition of oocyte maturation (11). Therefore, it seems that EGF cannot induce oocyte maturation by overcome tyrosine kinase inhibitors pathways of cAMP (31). 

An increase of ROS and a decrease of TAC during *in vitro* culture in both DOs and COCs up to 36 hours have been detected in the present study. This is in agreement with other investigations which indicated OS was increased during cultivation period (14,15). However, ROS production of *in vitro* matured COC did not change up to 18 hours. It seems that activity of cumulus cells have been changed during IVM process of COCs, which in turn, led to conserve ROS concentration at the basal level. With respect to increase the number of cumulus cells during IVM of COCs, it is appeared that ROS production per cumulus cell has been declined. On the other hand, ROS concentration of *in vitro* matured DOs increased at 18 hours which is inconsistent with results of Cetica et al. (19), who showed ROS production did not change during bovine DOs IVM. It seems that, the conflicting result was due to differences in experimental strategies and animal species. 

Results of this study indicated that TAC level decreased in DOs after 18 hours of culture period while it did not change in COCs groups. Oocytes have their own enzymatic antioxidant activity which was attributed to expressed genes encoding antioxidant enzymes. Although, the COCs antioxidant capacity mostly dependent on cumulus cells (19,32). It is not known that this system of cumulus cells and oocytes could remain constant until the end of biphasic cultivation period since TAC (18) levels decreased at 36 hours in both *in vitro* cultured DOs and COCs. 

In order to reduce OS, supplementing culture medium with antioxidants has been broadly used (17,18,33), although the advantages of adding antioxidants in oocyte maturation medium still remains controversial (15). The results of the present study showed that ALA as a potent antioxidant could not affect the maturation rates of both COCs and DOs in one and two steps *in vitro* culture manner. However, in the ALA treated groups, ROS and TAC levels were decreased and increased, respectively. It was demonstrated that supplemented medium with non-enzymatic and enzymatic antioxidant improves the developmental competence of *in vitro* matured bovine oocyte (33). Whereas, it was shown that antioxidants restrain resumption of meiosis, which may show a role of ROS in oocyte maturation (34). On the other hand, it was indicated that OS induces meiotic arrest (35). The inconsistent results may be due to the dose and types of antioxidants and animal species have been used. For example, supplemented maturation medium with cysteamine, cysteine, and β-mercaptoethanol could improve the rate of porcine embryo production (36,37), while β-mercaptoethanol, superoxide dismutase, or ascorbic acid had no optimistic effect on subsequent development on bovine oocytes maturation medium (38). It has been shown that ALA improves developmental competence of mouse isolated pre-antral follicles somewhat via decreasing and increasing ROS and TAC levels, respectively (17) , however, in the present study ALA treated groups in one or two step culture manner did not show any significant difference with untreated respective groups. It is appeared that, these inconsistency results are related to the duration of culture period because duration of cultivation period of isolated pre-antral follicle was 12 days while maximum duration of oocyte *in vitro* culture was 36 hours. In this regard, it has been shown that ROS production was increased during cultivation period (17,18). Also, regarding to ability of oocytes and cumulus cells to express genes encoding antioxidant enzymes (19,32) may explains why adding an antioxidant could not improve IVM rates of both COCs and DOs. In addition, it was revealed that certain levels of ROS during IVM may be beneficial and play a crucial role in the induction of oocyte maturation which in turn improve embryo production rates (39). 

## Conclusion

ALA supplemented maturation medium did not had a significant effect on IVM rates of both COCs and DOs, although it decreased ROS and increased TAC levels in two step *in vitro* matured oocytes. Furthermore, two step IVM manner with meiotic inhibitors was more effective on improvement of DOs maturation rates than COCs. 
